# A longitudinal study of structural brain network changes with normal aging

**DOI:** 10.3389/fnhum.2013.00113

**Published:** 2013-04-03

**Authors:** Kai Wu, Yasuyuki Taki, Kazunori Sato, Haochen Qi, Ryuta Kawashima, Hiroshi Fukuda

**Affiliations:** ^1^Department of Nuclear Medicine and Radiology, Institute of Development, Aging and Cancer, Tohoku UniversitySendai, Japan; ^2^Department of Biomedical Engineering, School of Materials Science and Engineering, South China University of TechnologyGuangzhou, China; ^3^Division of Medical Image Analysis, Department of Community Medical Supports, Tohoku Medical Megabank Organization, Tohoku UniversitySendai, Japan; ^4^Division of Developmental Cognitive Neuroscience, Institute of Development, Aging and Cancer, Tohoku UniversitySendai, Japan; ^5^Smart Ageing International Research Centre, Institute of Development, Aging and Cancer, Tohoku UniversitySendai, Japan; ^6^Department of Functional Brain Imaging, Institute of Development, Aging and Cancer, Tohoku UniversitySendai, Japan

**Keywords:** structural brain network, economical small-world, normal aging, longitudinal study, regional gray matter volume

## Abstract

The aim of this study was to investigate age-related changes in the topological organization of structural brain networks by applying a longitudinal design over 6 years. Structural brain networks were derived from measurements of regional gray matter volume and were constructed in age-specific groups from baseline and follow-up scans. The structural brain networks showed economical small-world properties, providing high global and local efficiency for parallel information processing at low connection costs. In the analysis of the global network properties, the local and global efficiency of the baseline scan were significantly lower compared to the follow-up scan. Moreover, the annual rate of change in local and global efficiency showed a positive and negative quadratic correlation with the baseline age, respectively; both curvilinear correlations peaked at approximately the age of 50. In the analysis of the regional nodal properties, significant negative correlations between the annual rate of change in nodal strength and the baseline age were found in the brain regions primarily involved in the visual and motor/control systems, whereas significant positive quadratic correlations were found in the brain regions predominately associated with the default-mode, attention, and memory systems. The results of the longitudinal study are consistent with the findings of our previous cross-sectional study: the structural brain networks develop into a fast distribution from young to middle age (approximately 50 years old) and eventually became a fast localization in the old age. Our findings elucidate the network topology of structural brain networks and its longitudinal changes, thus enhancing the understanding of the underlying physiology of normal aging in the human brain.

## Introduction

Recent advances in generating a network map of the human brain, known as the human connectome, provided new insights into structural and functional connectivity patterns of the human brain (Sporns et al., 2005; Bullmore and Bassett, 2011; Sporns, 2011a,b). The quantitative analysis of the structural and functional systems of the human brain, based largely on graph theory, reveal the topological properties of complex networks, such as economical small-world properties, highly connected hubs, and modularity (Bullmore and Sporns, 2009; He and Evans, 2010; Wig et al., 2011). Prodigious efforts in the study of the human connectome have greatly expanded our knowledge of the topological principles of brain network organization in the healthy, developing, aging, and diseased brains (Bassett and Bullmore, 2009; Uddin et al., 2010; Lo et al., 2011; Xia and He, 2011; Xie and He, 2011; Greicius and Kimmel, 2012; Sun et al., 2012).

It has been well-established that advanced aging is accompanied by cognitive decline, even in the absence of disease. Cognitive deficits in normal aging might arise from anatomical changes in specific brain regions or alterations of the structural and functional associations between distinct brain regions (Andrews-Hanna et al., 2007). Normal aging has been proven to be associated with changes in both functional (Achard and Bullmore, 2007; Meunier et al., 2009; Wang et al., 2010, 2012; Meier et al., 2012; Spreng and Schacter, 2012) and structural (Gong et al., 2009; Montembeault et al., 2012; Wu et al., 2012; Zhu et al., 2012) brain networks. However, these findings were revealed by cross-sectional studies, and few studies using a longitudinal design have been applied to investigate human brain networks with normal aging. Several Alzheimer's disease Neuroimaging Initiative (ADNI) studies have shown longitudinal changes in default mode network (DMN) regions, including the medial temporal lobe and posterior cingulate cortex (PCC), as patients progress into Alzheimer's disease (AD) and through its later stages (Risacher et al., 2010; Li et al., 2012). Thus, we hypothesized that significant longitudinal changes might occur in the topological properties of structural brain networks with normal aging.

By applying a longitudinal design over 6 years in a large number of healthy subjects aged 21–80, our previous studies have indicated the following: significant correlations between the annual percentage change in the ratio of gray matter and the age at baseline (Taki et al., 2011a), as well as significant correlations between the annual rate of regional gray matter volume change in many brain regions and the age at baseline (Taki et al., 2012a). In the present study, we aimed to investigate structural brain networks with normal aging by applying the above-mentioned longitudinal design. Structural brain networks have been constructed from inter-regional correlation of morphological measurements [e.g., cortical thickness (He et al., 2007), regional gray matter volume (RGMV) (Bassett et al., 2008), and surface area (Sanabria-Diaz et al., 2010)] in structural magnetic resonance imaging (sMRI) data. Recently, many studies have investigated the topological organization of structural brain networks in health [e.g., healthy subjects with normal aging (Montembeault et al., 2012; Wu et al., 2012; Zhu et al., 2012)] and disease [e.g., AD (He et al., 2008), multiple sclerosis (He et al., 2009), schizophrenia (Bassett et al., 2008), and breast cancer (Hosseini et al., 2012)]. In this study, we divided 380 healthy subjects into 29 age-specific groups using a sliding boxcar grouping ordered by baseline age. A structural brain network consisting of 90 regions was constructed by computing the correlation matrix of the RGMV across subjects within each age group in both the baseline and follow-up scans. We then computed both global and regional network properties in the structural brain networks and compared their differences between baseline and follow-up. Finally, to characterize the longitudinal changes of structural brain networks with normal aging, the correlations between the baseline age and the annual rate of change in both global and regional network properties were analyzed.

## Materials and methods

### Subjects

The subjects were normal, community-dwelling Japanese subjects recruited by the Aoba Brain Imaging Project (Sato et al., 2003). Subject recruitment was described previously (Taki et al., 2011a,b,c, 2012a,b). Briefly, we performed longitudinal follow-up (Aoba2) scans of 442 subjects who were selected from 1604 participants in the baseline (Aoba1) scan. In both the baseline and follow-up scans, we excluded those subjects who had a past or present history of malignant tumors, head traumas, cerebrovascular diseases, epilepsy, or psychiatric diseases. After the interview, brain MR images were obtained from each subject. The MR images were inspected by 2–3 well-trained radiologists. Images with any of the following findings were excluded from this study: head injuries, brain tumors, hemorrhage, major and lacunar infarctions, or moderate to severe white matter hyperintensities. Thus, the final sample consisted of 380 participants (157 men/223 women). The mean ± standard deviation (SD) interval between baseline and follow-up was 7.41 ± 0.54 years (range, 6.1–9.0). The mean ± SD age of the participants at baseline was 51.1 ± 11.7 years old (range, 21–80).

A total of 11 subjects (mean age = 65.3 years; range, 57.7–73.4 years at follow-up; 3 men/8 women) were scanned twice on the same day to obtain an estimation of the measurement reliability. We observed no significant differences in the gray matter volume or intracranial volume between the baseline and follow-up scans. The details of the measurement reliability are reported elsewhere (Taki et al., 2011a).

After a full explanation of the purpose and procedures of the study, written informed consent according to the Declaration of Helsinki (1991) was obtained from each subject prior to MRI scanning. Approval for these experiments was obtained from the institutional review board of Tohoku University.

### Image acquisition

All images were collected using the same 0.5-T MR scanner (Signa contour; GE-Yokogawa Medical Systems, Tokyo, Japan) for both the baseline and follow-up studies. The scanner was routinely calibrated using the same standard GE phantom between baseline and follow-up. During the course of this study, no major hardware upgrade occurred. At baseline and follow-up, all subjects were scanned with identical pulse sequences: 124 contiguous, 1.5-mm-thick axial planes of three-dimensional T1-weighted images (spoiled gradient recalled acquisition in steady state: repetition time, 40 ms; echo time, 7 ms; flip angle, 30; voxel size, 1.02 mm × 1.02 mm × 1.5 mm).

### Measurements of regional gray matter volume

After the image acquisition, the RGMV for each subject was measured using statistical parametric mapping 2 (SPM2) (Wellcome Department of Cognitive Neurology, London, UK) (Friston et al., 1995) in Matlab (MathWorks, Natick, MA). First, the T1-weighted MR images were transformed to the same stereotactic space by registering each of the images to the ICBM 152 template (Montreal Neurological Institute, Montreal, Canada), which approximates the Talairach space (Jean Talairach, 1988). Then, tissue segmentation from the raw images to the gray matter, white matter, cerebrospinal fluid space, and non-brain tissue was performed using the SPM2 default segmentation procedure. We applied these processes using the “cg_vbm_optimized” MATLAB function (http://dbm.neuro.uni-jena.de/vbm.html). WFU_PickAtlas software was employed to label the regions in the gray matter images, providing a method for generating ROI masks based on the Talairach Daemon database (Lancaster et al., 2000; Maldjian et al., 2003, 2004). To calculate the regional gray matter volume (RGMV) for each subject, we parcellated the entire gray matter into 45 separate regions for each hemisphere (90 regions in total, see Table 1) defined by the Automated Anatomical Labeling (AAL) atlas (Tzourio-Mazoyer et al., 2002).

**Table 1:** **Regions of interest included in AAL-atlas**.

**Lobes**	**Regions**	**Abbreviations**	**Lobes**	**Regions**	**Abbreviations**
Frontal	Precentral gyrus	PreCG	Temporal	Hippocampus	HIP
	Superior frontal gyrus (dorsal)	SFGdor		Parahippocampal gyrus	PHG
	Orbitofrontal cortex (superior)	ORBsup		Amygdala	AMYG
	Middle frontal gyrus	MFG		Fusiform gyrus	FFG
	Orbitofrontal cortex (middle)	ORBmid		Heschl gyrus	HES
	Inferior frontal gyrus (opercular)	IFGoperc		Superior temporal gyrus	STG
	Inferior frontal gyrus (triangular)	IFGtriang		Temporal pole (superior)	TPOsup
	Orbitofrontal cortex (inferior)	ORBinf		Middle temporal gyrus	MTG
	Rolandic operculum	ROL		Temporal pole (middle)	TPOmid
	Supplementary motor area	SMA		Inferior temporal gyrus	ITG
	Olfactory	OLF	Occipital	Calcarine cortex	CAL
	Superior frontal gyrus (medial)	SFGmed		Cuneus	CUN
	Orbitofrontal cortex (medial)	ORBmed		Lingual gyrus	LING
	Rectus gyrus	REC		Superior occipital gyrus	SOG
	Anterior cingulate gyrus	ACG		Middle occipital gyrus	MOG
	Middle cingulate gyrus	MCG		Inferior occipital gyrus	IOG
Parietal	Posterior cingulate gyrus	PCG	Subcortical	Caudate	CAU
	Postcentral gyrus	PoCG		Putamen	PUT
	Superior parietal gyrus	SPG		Pallidum	PAL
	Inferior parietal lobule	IPL		Insula	INS
	Supramarginal gyrus	SMG		Thalamus	THA
	Angular gyrus	ANG			
	Precuneus	PCUN			
	Paracentral lobule	PCL			

### Construction of structural brain networks

**Figure 1:**
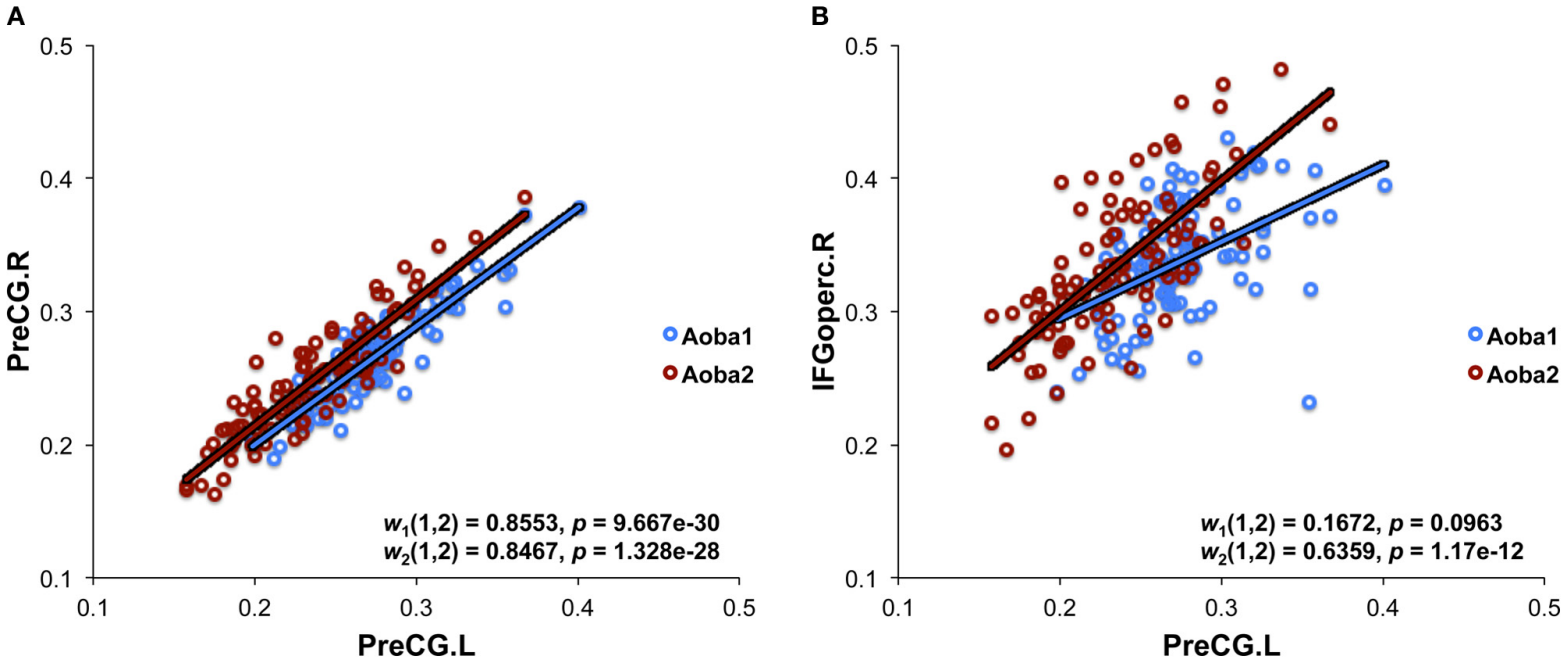
**Structural connectivity derived from the measurement of regional gray matter volume. (A)** The structural connectivity between the bilateral precentral gyrus (PreCG). **(B)** The structural connectivity between left PreCG and the left opercular part of the inferior frontal gyrus (IFGoperc). The plots indicate Pearson's correlation coefficients [*w*_*N*_(*i, j*)] between two brain regions (*i* and *j*) using the measurement of regional gray matter volume, which was corrected by a linear regression analysis to remove the effects of total gray matter volume, age, sex, and age-by-sex interaction. The data from both the baseline (Aoba1, *N* = 1) and follow-up (Aoba2, *N* = 2) scans are shown.

We applied the methodology described in our previous studies (Wu et al., 2011, 2012) to construct structural brain networks. Briefly, we computed a correlation matrix using the measurement of RGMV across a group of subjects. In this study, we created 29 age groups using a sliding boxcar grouping (Fair et al., 2009) in the order of baseline age (i.e., Group1: subjects 1–100, Group2: subjects 11–110, Group3: subjects 21–120, … Group 29: subjects 281–380). Similarly, 29 age groups in the follow-up scan were also created, which corresponded to the age groups in the baseline scan. For each age group, a linear regression analysis was performed on the RGMV to remove the effects of the total gray matter volume, age, sex, and age-by-sex interaction. Thus, the residuals of this regression were employed as the substitute for the raw RGMV and denoted as the corrected RGMV (cRGMV). We then computed the Pearson correlation coefficient between cRGMV across 100 subjects included in one group to construct an interregional correlation matrix (*N* × *N*, where *N* is the number of gray matter regions; here, *N* = 90). Each element of the correlation matrix represents the structural connectivity between two regions. For example, the bilateral precentral gyrus (PreCG) showed strong correlations in Group 1 in both the baseline and follow-up scans (Figure 1A), indicating high connectivity between the same region in the bilateral hemispheres; however, the correlation between the left PreCG and the left opercular part of the inferior frontal gyrus (IFGoperc) in Group 1 was stronger in the follow-up scan compared to the baseline scan (Figure 1B). A correlation matrix (*r*_*ij*_, *N* × N) can be converted to a weighted and undirected network *G* using a cost threshold approach (*t*, 0 < *t* < 1), which can normalize all networks to have the same number of edges or wiring cost and, thus, provide an avenue to detect changes in topological organization with aging (Achard and Bullmore, 2007).

$$G(i,\;j)=\{\begin{array}{l} 1,\;\left|r_{ij}\right|\geq r_{t}\\ 0,\;\left|r_{ij}\right|<r_{t} \end{array}$$

Finally, we constructed a structural brain network for each of the 29 age groups in both the baseline and follow-up scans.

### Graph theoretical analysis

To ensure that the resulting brain networks are sparse, fully connected, and distinguishable from degree-matched random and regular networks, we adopted a range of cost thresholds (0.11 ≤ t ≤ 0.25, step = 0.01) to calculate the topological properties of structural brain networks (Bassett et al., 2008; Liu et al., 2008; Wang et al., 2009b; Wu et al., 2012). Small-world efficiency metrics (local efficiency, *LE*, and global efficiency, *GE*) were computed to characterize the global network properties of the structural brain networks. The node strength (*NS*) was used to examine regional nodal properties because of its high test-retest reliability (Wang et al., 2011). Here, both global and regional network metrics are briefly described as follows (Rubinov and Sporns, 2010) and were calculated using the Brain Connectivity Toolbox (http://www.brain-connectivity-toolbox.net).

The global efficiency of the graph *G* can be computed as (Latora and Marchiori, 2001):

$$GE(G)=\frac{1}{N(N-1)}\sum_{i\;\neq \;j\;\in \;G}\frac{1}{d_{ij}},$$

where *d*_*ij*_ is the shortest path length between nodes *i* and *j*. The path length between nodes *i* and *j* is defined as the sum of the edge lengths along this path, where each edge's length was obtained by computing the reciprocal of the edge weight, 1/*w*_*ij*_. Thus, the shortest path length *d*_*ij*_ is the length of the path with the shortest length between nodes *i* and *j*. The local efficiency of the graph *G* is defined as (Latora and Marchiori, 2001):

$$LE(G)=\frac{1}{N}\sum_{i\;\in \;G}GE(Gi),$$

where *GE*(*G*_*i*_) is the global efficiency of *G*_*i*_, the subgraph of the neighbors of node *i*. The small-world efficiency metrics (*GE* and *LE*) of real brain networks were compared with 1000 random networks (*G*_rand_) that preserved the degree and weight distributions of real networks (Maslov and Sneppen, 2002). A real brain network is considered to be a small-world network if it shows similar global efficiency but much higher local efficiency than its matched random networks (Latora and Marchiori, 2001).

The node strength (*NS*_*i*_) for a given node *i* is defined as the sum of all of the edge weights between this node and all of the other nodes in the network. Regions with a high nodal strength indicate high interconnectivity with other regions.

Regarding the structural brain network for each age group, we averaged the global and regional network metrics (*LE*, *GE*, and *NS*) over the range of cost thresholds (0.11 ≤ t ≤ 0.25) to obtain the summary network metrics (Bassett et al., 2008). To investigate the longitudinal changes of network properties, the annual rate of change in the summary network metrics (*ARC_X*) was defined as:

$$ARC\_X=\frac{X_{2}-X_{1}}{{\text{Age}}_{2}-{\text{Age}}_{1}},$$

where *X*_1_ and *X*_2_ are the summary network metrics at baseline and follow-up, respectively; and Age_1_ and Age_2_ are the mean age of 100 subjects included in the age group at baseline and follow-up, respectively. The *ARC_X* value indicates the differences in summary network metrics between the baseline and follow-up scans, normalized by the interval of age.

### Statistical analysis

To analyze the differences in the summary global network properties (e.g., *LE* and *GE*) of the same age group between two scans (e.g., Group 1 at baseline vs. Group 1 at follow-up), a non-parametric permutation test method was applied (Bullmore et al., 1999; He et al., 2008; Wu et al., 2012). Moreover, a paired *t*-test was performed to determine whether there were significant longitudinal changes in each summary network metric (*LE*, *GE*, and *NS*) between all age groups at baseline and those at follow-up. To evaluate correlations between the longitudinal changes in network properties and the baseline age, we performed multiple linear regression analyses with the annual rate of change in the summary network metrics as the dependent variables and the baseline age as the independent variable. Here, three multiple linear regressions (Model I, II, and III) modeling mean value, age, age^2^, and age^3^ as predictors were applied to detect the linear, quadratic, and cubic changes with the baseline age. We then determined the best model among the three regressions using Akaike's information criterion (AIC) (Akaike, 1974).

(I) $$ARC\_X=\text{mean}+a\times {\text{Age}}_{1}+e$$

(II) $$ARC\_X=\text{mean}+a_{1}\times {\text{Age}}_{1}+a_{2}\times {\text{Age}}_{1}^{2}+e$$

(III) $$ARC\_X=\text{mean}+a_{1}\times {\text{Age}}_{1}+a_{2}\times {\text{Age}}_{1}^{2}+a_{3}\times {\text{Age}}_{1}^{3}+e$$

For the regression analysis of regional nodal property, we only included regions with significant differences in the summary regional network metric (e.g., *NS*) between the baseline and follow-up scans by the paired *t*-test (*p* < 0.05, FDR-corrected).

## Results

### Economical small-world properties and longitudinal changes

**Figure 2:**
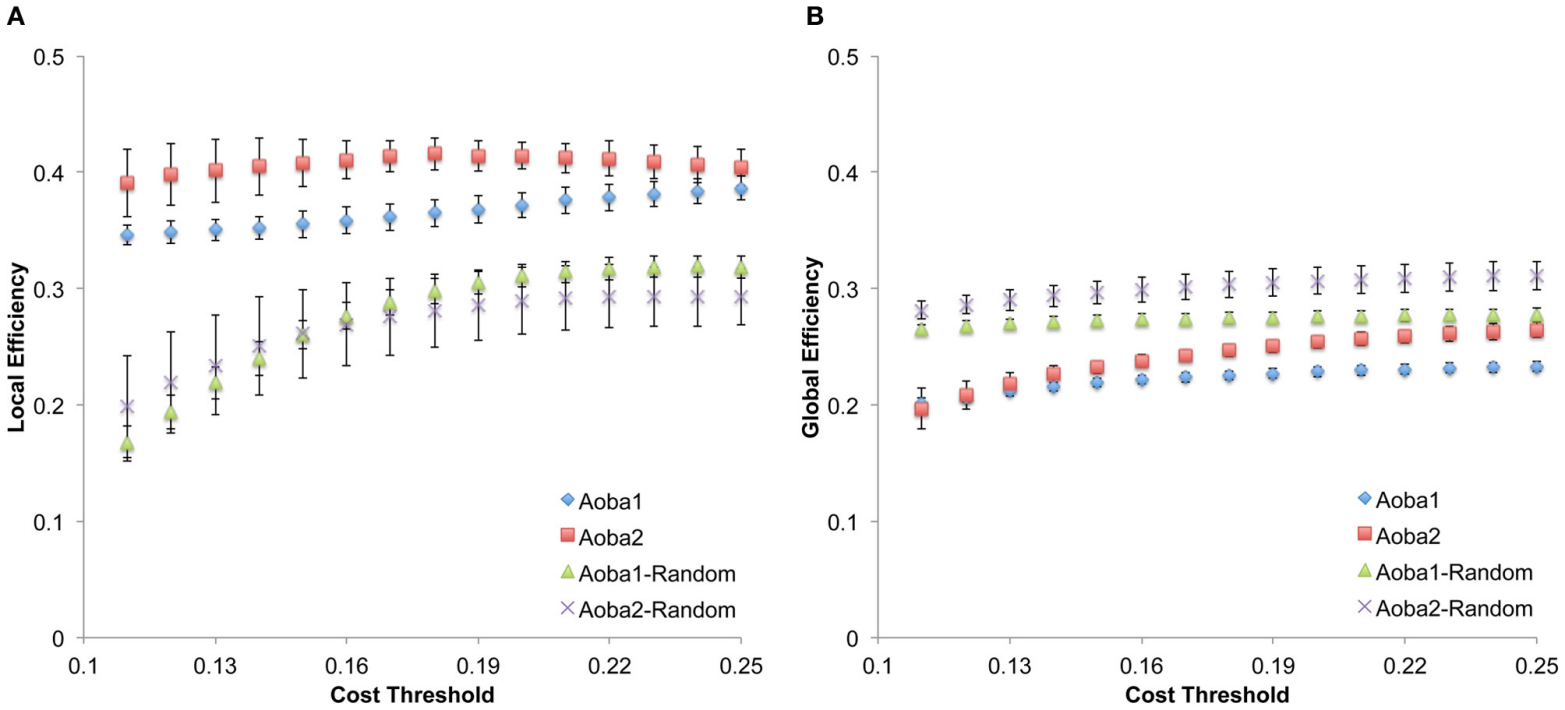
**Small-world efficiency properties in structural brain networks. (A)** Local efficiency calculated under the cost threshold range of 0.11–0.25. **(B)** Global efficiency calculated under the cost threshold range of 0.11–0.25. Aoba1-Random and Aoba2-Random correspond to the matched random networks for the structural brain network in Aoba1 and Aoba2, respectively.

**Figure 3:**
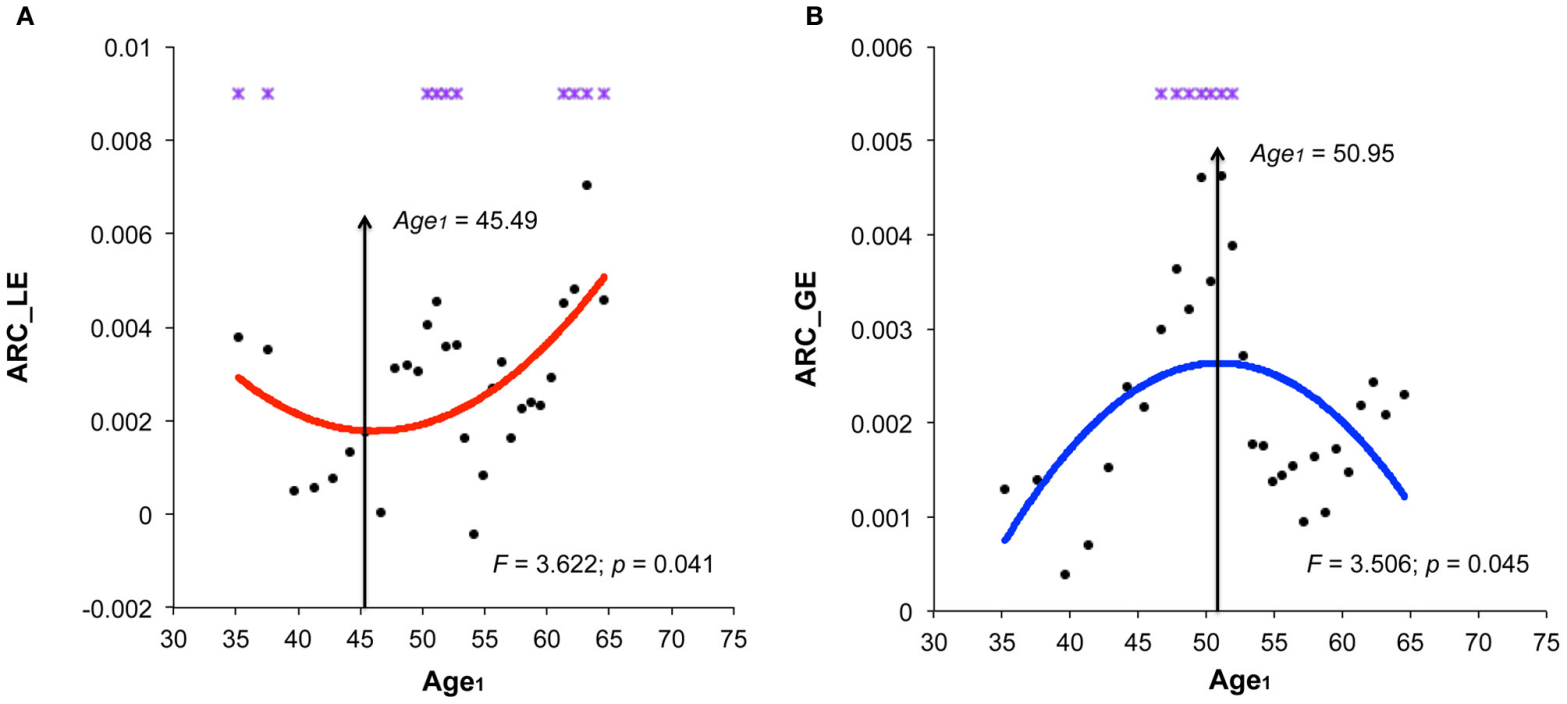
**Significant correlations between the annual rate of change in small-world efficiency and the baseline age. (A)** Significant positive quadratic correlations between the annual rate of change in local efficiency (*ARC_LE*) and the baseline age (Age_*1*_), peaked at the baseline age of 45.49 years. **(B)** Significant negative quadratic correlations between the annual rate of change in global efficiency (*ARC_GE*) and the baseline age, peaked at the baseline age of 50.95 years. Note that significant differences (*p* < 0.05) in the summary global network properties of the same age group between two scans by the nonparametric permutation test are indicated by violet stars.

The structural brain networks of the age-specific groups exhibited economical small-world properties, showing higher local efficiency but similar global efficiency compared to the matched random networks (Latora and Marchiori, 2001). This finding is illustrated in Figure 2, where we plot the local and global efficiency of the structural brain networks of the age-specific groups from both the baseline and follow-up scans against those of the matched random networks. Moreover, significant differences (a non-parametric permutation test; *p* < 0.05) in the summary local efficiency were found in several age groups across the baseline age but those in the summary global efficiency were found in the middle age groups (Figure 3). For all age groups, the structural brain networks from the baseline scan showed significantly lower local efficiency (a paired *t*-test; *t*-value = 8.446; *p* < 10^−4^) and global efficiency (a paired *t*-test; *t*-value = 10.478; *p* < 10^−4^) compared to those from the follow-up scan. The annual rate of change in local efficiency (*ARC_LE*) and global efficiency (*ARC_GE*) showed a positive quadratic (*F*-value = 3.622, *p* = 0.041) and a negative quadratic (*F*-value = 3.506, *p* = 0.045) correlation with the baseline age, respectively (Figures 3A,B). The curvilinear correlations peaked at the baseline ages of 45.49 years and 50.95 years, respectively.

### Regional nodal properties and longitudinal changes

**Figure 4:**
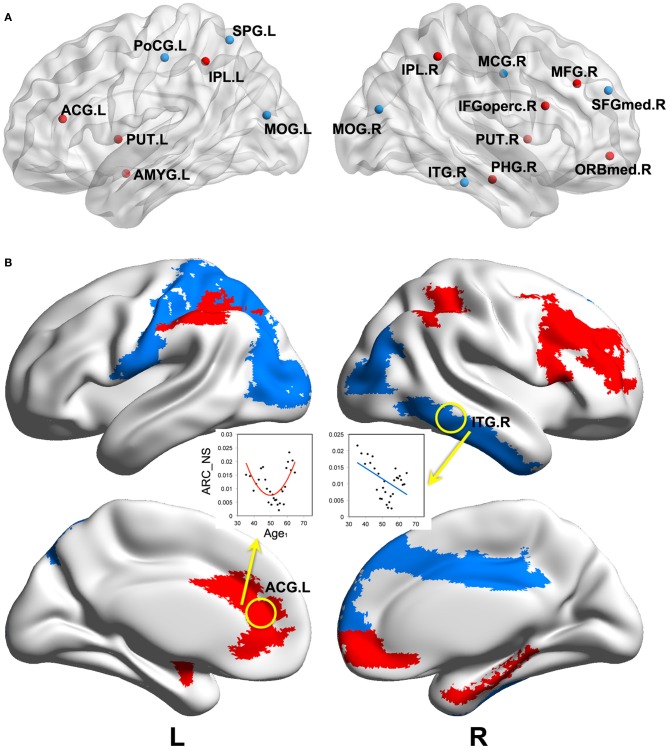
**Significant correlations between the annual rate of change in node strength and the baseline age.** Significant correlations (*p* < 0.05) are visualized in anatomical space **(A)** and mapped onto the cortical surface **(B)**. Negative linear and positive quadratic correlations are indicated by blue and red colors, respectively. *ARC_NS*: the annual rate of change in node strength; Age_1_: the baseline age. Abbreviations are shown in Table 1.

We found significant correlations between the annual rate of change in node strength (*ARC_NS*) and the baseline age in many brain regions, which showed significant differences in the node strength (a paired *t*-test; *p* < 0.05, FDR-corrected) between the baseline and follow-up scans. Specifically, significant negative correlations (*p* < 0.05) between the *ARC_NS* and the baseline age were found in several brain regions that were primarily related to the visual system [e.g., the bilateral middle occipital gyrus (MOG) and right inferior temporal gyrus (ITG)] and the motor/control system [e.g., the left postcentral gyrus (PoCG), left superior parietal gyrus (SPG), right medial part of superior frontal gyrus (SFGmed), and right middle cingulate gyrus (MCG)] (Table 2, Figure 4). Significant positive quadratic correlations (*p* < 0.05) between the *ARC_NS* and the baseline age were found in several brain regions that were mainly associated with the default-mode system [e.g., the left anterior cingulate gyrus (ACG) and right medial part of the orbitofrontal cortex (ORBmed)], the attention system [e.g., the right middle frontal gyrus (MFG), right IFGoperc, and bilateral inferior parietal lobule (IPL)], and the memory system [e.g., the right parahippocampal gyrus (PHG), left amygdala (AMYG), and bilateral putamen (PUT)] (Table 3, Figure 4); the significant positive quadratic correlations peaked at a baseline age of 51.17–54.87 years (Table 3). The regional nodal properties of these brain regions were visualized in anatomical space (Figure 4A) and mapped onto the cortical surface (Figure 4B) using the BrainNet Viewer (http://www.nitrc.org/projects/bnv/).

**Table 2:** **Significant negative linear correlation between the annual rate of change in node strength and the baseline age**.

**System**	**Lobe**	**Class**	**Abbreviation**	***F* value**
Visual	Occipital	Association	MOG.L	10.416
	Occipital	Association	MOG.R	11.194
	Temporal	Association	ITG.R	21.121
Motor/control	Parietal	Primary	PoCG.L	13.561
	Parietal	Association	SPG.L	14.523
	Frontal	Association	SFGmed.R	17.964
	Frontal	Paralimbic	MCG.R	18.481

*The level of significance was set at p < 0.05*.

**Table 3:** **Significant positive quadratic correlation between the annual rate of change in node strength and the baseline age**.

**System**	**Lobe**	**Class**	**Abbreviation**	***F* value**	**Peak at the baseline age**
Default-mode	Frontal	Paralimbic	ACG.L	8.859	51.17
	Frontal	Paralimbic	ORBmed.R	25.981	53.40
Attention	Frontal	Association	MFG.R	13.169	54.87
	Frontal	Association	IFGoperc.R	18.572	54.15
	Parietal	Association	IPL.L	12.596	51.93
	Parietal	Association	IPL.R	12.521	54.23
Memory	Temporal	Paralimbic	PHG.R	12.612	53.51
	Temporal	Paralimbic	AMYG.L	33.272	52.03
	Subcortical	Subcortical	PUT.L	14.899	52.59
	Subcortical	Subcortical	PUT.R	16.783	53.62

*The level of significance was set at p < 0.05*.

## Discussion

To our knowledge, this is the first study to investigate longitudinal changes in the topological organization of structural brain networks in a large number of healthy individuals. We found that the structural brain networks of age-specific groups exhibit economical small-world properties. *ARC_LE* and *ARC_GE* showed significant curvilinear correlations with the baseline age, with a peak at the baseline age of approximately 50. Our results also revealed significant correlations between the *ARC_NS* and the baseline age in many brain regions. Structural brain networks develop into a more distributed organization from young to middle age (approximately 50 years old) and then achieve a localized organization with substantial alterations in old age. Thus, revealing longitudinal changes in the topological properties of structural brain networks may enhance our understanding of the physiology underlying normal aging in the human brain.

### Economical small-world properties and longitudinal changes

In this study, the structural brain networks derived from measurements of RGMV in all age-specific groups exhibited the key properties of economical small-world organization. An economical small-world network can provide a topological substrate for both locally specialized processing in the neighborhoods of highly clustered nodes and globally distributed processing on a highly efficient network with short characteristic path lengths (Sporns and Zwi, 2004; Stam, 2004; Achard et al., 2006; Achard and Bullmore, 2007). Our finding of high global and local efficiency in the structural brain networks is consistent with the results of previous functional and structural brain networks studies (He et al., 2007, 2008, 2009; Bassett et al., 2008, 2009; Wang et al., 2009b; Khundrakpam et al., 2012; Wu et al., 2012; Zhu et al., 2012).

We also noted longitudinal changes in small-world efficiency metrics of the structural brain networks. Several age groups in the follow-up scan showed significant higher values in local or global efficiency compared to those in the baseline scan. Moreover, the differences in both local and global efficiency between two scans varied across the age groups and showed significant correlations with the baseline age. *ARC_LE* and *ARC_GE* showed a U-curve and an inverted-U curve trajectory with the baseline age, respectively. In particular, the trajectories of *ARC_LE* and *ARC_GE* peaked at a baseline age of 45.49 and 50.95 years, respectively. These results are consistent with our previous cross-sectional study findings, in which the local and global efficiency showed U-curve and inverted-U curve tendencies, respectively, in young (18–40 years), middle (41–60 years), and old age (61–80 years) groups (the subjects used were from the same dataset of the baseline scan in this study) (Wu et al., 2012).

The longitudinal changes in local and global efficiency could be divided into two processes based on the peaks. First, the period from young to middle age (approximately 50 years old) showed decelerated increases in local efficiency and accelerated increases in global efficiency, indicating a fast distribution in the middle age. This period might reflect a maturation process in the structural brain network. A previous study demonstrated that the organization of multiple functional networks shifts from a local anatomical emphasis in children to a more distributed architecture in young adults, indicating the maturation process of the functional systems (Fair et al., 2009). A more recent study on structural brain networks constructed from the measurement of cortical thickness also indicated a more distributed configuration in late childhood, accompanied by significant increases in global efficiency but decreases in local efficiency (Khundrakpam et al., 2012). In addition, white matter plays a vital role in the efficient transfer of information between gray matter regions. Our previous longitudinal study of a large number of healthy subjects (the same datasets of both the baseline and follow-up scans in this study) demonstrated that the white matter ratio increased until approximately age 50 and then decreased in both men and women (Taki et al., 2011a). Several previous studies also indicated that white matter volume seems to increase until the middle age of approximately 45 years and decrease thereafter (Bartzokis et al., 2001; Sowell et al., 2003). Increases in the white matter represent maturational changes, such as myelination that continue until middle adulthood and may, therefore, provide evidence of the maturation of structural brain networks.

Second, the period from middle (approximately 50 years old) to old age showed an accelerated increase in local efficiency and a decelerated increase in global efficiency, leading to a fast localization in the old age. The changes over this period might reflect a degenerative process in the structural brain network with advanced aging. A recent study demonstrated that the structural brain networks in an older cohort (mean age = 66.6 years, range 64–68) had lower global efficiency but higher local efficiency, revealing a more localized configuration compared to the younger cohort (mean age = 46.7 years, range 44–48) (Zhu et al., 2012). Using a sample of 342 healthy individuals aged 72–92 years, a previous DTI tract-derived connectivity study indicated that the global efficiency of the structural brain networks decreased significantly with older age (Wen et al., 2011). It is important to note that a regular configuration with less global integration upsets the optimal balance of a small-world network and is related to many neurological and psychiatric disorders described as dysconnectivity syndromes (Catani and ffytche, 2005). Several previous studies have reported a regular configuration or a reduction in the global efficiency of brain networks in patients with diseases such as AD and amnestic mild cognitive impairment (aMCI, the prodromal stage of AD), providing further support for the characterization of AD and aMCI as dysconnectivity syndromes and indicating the functional basis of cognitive deficits (Stam et al., 2007; He et al., 2008; Bai et al., 2012; Zhao et al., 2012; Wang et al., 2013). Therefore, we speculate that advanced aging is associated with a high risk for dysconnectivity syndromes.

### Regional nodal properties and longitudinal changes

Node strength measures the interconnectivity of a node with other regions and can be used to determine the relative importance of a node within a network. We identified significant correlations between the *ARC_NS* and the baseline age in many brain regions, mainly consisting of recently evolved association (9/17) and primitive limbic/paralimbic (5/17) regions. Association regions contribute to the integrity of multiple functional systems such as the attention and memory systems, while limbic/paralimbic regions are highly interconnected with the prefrontal regions and subcortical regions and are mainly involved in emotional processing and the maintenance of a conscious state of mind (Mesulam, 1998). Thus, our results support the view that age-related changes are mainly a characteristic of the association cortex rather than the primary cortex (Albert and Knoefel, 1994).

The brain regions showing significant negative correlations with the baseline are primarily involved in the visual and motor/control systems. A previous study of the structural brain networks in elderly subjects using DTI data demonstrated significant positive correlations between the regional nodal efficiency and visuospatial, processing speed, and executive functions in many cortical regions (Wen et al., 2011). Therefore, we speculate that our findings of the decreases of *ARC_NS* with the baseline age in the visual and motor/control systems might be related to the decline of these functions with normal aging. It is well-known that visual abilities decline during normal (non-pathological) aging, and older individuals tend to have reduced visual acuity and contrast sensitivity (Spear, 1993; Owsley, 2011). A recent study using event-related potentials (ERPs) also found that visual acuity declined as a function of age when young adults (18–32 years), young–old adults (65–79 years), and old–old adults (80+ years) performed a visual processing task involving selective attention to color (Daffner et al., 2012). Moreover, normal aging-related degeneration in the brain is accompanied by reduced force control, progressive slowness, and impaired motor ability (Roos et al., 1997; Smith et al., 1999; Krampe, 2002). Worsened task performance (e.g., slower speed with increasing memory load) in old adults (mean age = 71.27) is associated with decreases in the functional network connectivity between components comprising the supplementary motor area and the middle cingulate gyrus and between the precuneus and the middle/superior frontal cortex (Steffener et al., 2012). A previous resting-state fMRI study indicated a significant decrease in the functional connectivity of the motor network in aged subjects (mean age = 61.8) compared to young subjects (mean age = 26.5 years) (Wu et al., 2007). A more recent fMRI study using a visual oddball task also indicated that elderly subjects (mean age = 63.9 years) showed a decrease in connectivity within the somatomotor network compared to younger subjects (mean age = 24.1 years) (Geerligs et al., 2012).

We also found significant quadratic correlations between the *ARC_NS* and the baseline age in many brain regions, predominately from frontal (4/10), temporal (2/10), parietal (2/10), and subcortical (2/10) areas. It is notable that the significant quadratic correlations peaked at a baseline age from 51.17 to 54.87 years. Thus, in these brain regions, the *ARC_NS* increased with the baseline age in the period from middle (approximately 50 years old) to old age. More importantly, the identified brain regions are mainly associated with the default-mode, attention, and memory systems. The scaffolding theory of aging and cognition (STAC) suggests that scaffolding is a normal process present across the lifespan that involves use and development of complementary, alternative neural circuits to achieve a particular cognitive goal and is protective of cognitive function in the aging brain (Park and Reuter-Lorenz, 2009). Thus, our results are in line with the STAC and suggest a compensation mechanism of structural brain network reorganization with advanced aging. It has been indicated that cognitive decline is associated with differences in the structure and function of the aging brain, and it has been suggested that increased activation is either caused by disruption, whether structural or functional, or is a compensatory response to such disruption (Hedden and Gabrieli, 2004; Persson et al., 2006; Grady, 2012). Previous findings from several studies on structural and functional brain networks also support this view. Many brain regions, primarily from the frontal and temporal lobes, show increases in regional nodal efficiency in structural brain networks (Gong et al., 2009). Several regions, mostly in the lateral occipital-parietal junction and the paralimbic/subcortical area, reveal increased node betweenness in old age (Wu et al., 2012). The decrease in visual memory and visuoconstructive functions is strongly associated with the age-dependent enhancement of functional connectivity in both temporal lobes (Schlee et al., 2012). However, a recent study showed reduced structural association in the high-order cognitive networks of older adults compared to young adults, while no differences were observed in the sensorimotor networks (Montembeault et al., 2012). The following possible reasons are given for the discrepancies between this finding and our results: only eight brain regions were included in the previous study, whereas the present study was a whole-brain analysis; furthermore, only a comparison between young (mean age = 23.5 ± 3.1 years) and old (mean age = 67.3 ± 5.9 years) age was analyzed in the previous study, neglecting the other comparisons (young vs. middle; middle vs. old). Moreover, most of the identified brain regions showing positive quadratic correlations with the baseline age are found to be altered in AD patients (Bai et al., 2012; Zhao et al., 2012; Wang et al., 2013). For example, several brain regions (e.g., ACG.L, ORBmed.R, IFGoperc.R, IPL.L, IPL.R, and PUT.R) in AD patients show significant increases in regional nodal properties (e.g., the regional local and global efficiency) (Zhao et al., 2012). Thus, these findings provide further evidence supporting the view that advanced aging confers a high risk for neurodegenerative diseases, such as AD.

### Methodology

Several methodological issues need to be addressed. First, structural brain networks can be constructed in two ways: (1) indirectly from inter-regional correlation of morphological measurements (e.g., cortical thickness, RGMV, and surface area) in sMRI data; (2) directly from characteristics of white matter fibers (e.g., fiber number, fractional anisotropy, apparent diffusion coefficient, or distance) in diffusion tensor imaging (DTI) data (Bassett and Bullmore, 2009; He and Evans, 2010; Lo et al., 2011; Xia and He, 2011). Although there is still no direct proof that correlations of morphological measurements across subjects are indicative of axonal connectivity via white matter tracts, strong correlations between brain regions known to be anatomically connected have been observed in previous optimized voxel-based morphometry studies (Mechelli et al., 2005; Pezawas et al., 2005). Moreover, a recent study indicated that approximately 35–40% of cortical thickness correlations showed convergent diffusion connections across the cerebral cortex and most of them were the positive thickness correlations (Gong et al., 2012). However, the authors also found that almost all of the negative correlations (>90%) did not have a matched diffusion connection, suggesting different mechanisms behind the positive and negative thickness correlations. Since we defined structural connectivity as the absolute value of correlation of RGMV in this study, the association between correlation of RGMV and diffusion connections should be investigated further in future studies. Second, previous studies indicate that different parcellation strategies affect the topological properties (e.g., the local efficiency, global efficiency, small-worldness, and modularity) of structural or functional brain networks (Wang et al., 2009a; Fornito et al., 2010; Zalesky et al., 2010). A previous study also indicates that regional volumes are positively correlated with their mutual information, which measures the functional connectivity between each region and the remaining brain regions (Salvador et al., 2008). Thus, variations in parcellation templates (e.g., AAL used in this study) may affect the network structure of the human brain; future studies should include comparisons of network topology with different parcellation templates. Third, because all of the subjects in this study were over 20 years old, young and adolescent subjects should be included in future studies of brain network development. Finally, further investigations will also examine longitudinal changes in the topological properties of the human brain network using different neuroimaging modalities, such as diffusion tensor imaging, functional MRI, and electroencephalography.

## Conclusion

In this study, we quantitatively analyzed the topological organization of structural brain networks using a longitudinal design over 6 years. Our results reveal economical small-world properties of structural brain networks and longitudinal changes in both global and regional network properties. The structural brain networks develop into a fast distribution at approximately the age of 50 and then transform into a fast localization with substantial alterations in old age. Our findings may contribute to understanding the mechanism of normal aging in the human brain and help to distinguish neurodegenerative diseases from normal aging.

### Conflict of interest statement

The authors declare that the research was conducted in the absence of any commercial or financial relationships that could be construed as a potential conflict of interest.
